# Challenges in diagnosing polyethylene glycol and polysorbate 80 allergies: implications for allergic reactions in COVID-19 mRNA vaccination program: experience from Qatar

**DOI:** 10.3389/falgy.2024.1502285

**Published:** 2025-01-07

**Authors:** Sami Aqel, Sherin Thalappil, Asaad Imameldin, Dalal Mudawi, Muna Al Maslamani, Abdullatif Al-Khal, Hassan Mobayed, Maryam Ali Al-Nesf, Tayseer Ibrahim

**Affiliations:** ^1^ Allergy and Immunology Division, Department of Medicine, Hamad Medical Corporation, Doha, Qatar; ^2^ Communicable Disease Center, Hamad Medical Corporation, Doha, Qatar

**Keywords:** allergy testing, anaphylaxis, excipients, COVID-19 vaccines, polyethylene glycol allergy, polysorbate allergy, Qatar

## Abstract

**Introduction:**

COVID-19 vaccination has been a key intervention in reducing the severity of symptoms; however, concerns about vaccine safety, particularly regarding allergic reactions, arose early on. Healthcare workers faced the challenge of addressing these concerns to ensure safe vaccine administration. This study aimed to review the practical aspects of using allergy skin testing for COVID-19 vaccine excipients in patients with a history of allergic reactions developed following mRNA COVID-19 vaccination.

**Methods:**

A retrospective chart review was conducted for patients who reported allergic reactions after the COVID-19 vaccine and underwent allergy skin testing for COVID-19 vaccine excipients in the Adult Allergy and Immunology Service at Hamad Medical Corporation, Doha, Qatar. The testing protocol, developed based on published data during the pandemic, included skin prick (SPT) and intradermal (ID) testing using medications containing polysorbate 80 and polyethylene glycol (PEG), the primary excipients in the COVID-19 vaccines suspected of triggering allergic responses.

**Results:**

Of the 88 patients reviewed, 38 reported different types of allergic reactions following mRNA COVID-19 vaccination, with the majority being female. Anaphylaxis was reported in 21.1% of the patients, while the remaining experienced less severe allergic reactions. All patients underwent SPT and ID testing with PEG and polysorbate 80. By SPT, two patients tested positive for PEG and none for polysorbate 80. By ID, seven tested positive for polysorbate 80 and one for PEG. Among patients who experienced anaphylaxis, 50% had positive allergy test results. Twenty-three percent of patients with negative test results could receive additional vaccine doses without adverse reactions.

**Conclusion:**

Managing patients with a history of allergic reactions to the COVID-19 vaccine is challenging, as the exact mechanisms and accurate and valid allergy testing are yet to be determined. In our cohort, most patients had mild allergic reactions following vaccination. Excipients' allergy skin testing has helped to reduce vaccine hesitancy despite its questionable utility in clinical practice.

## Introduction

During the COVID-19 pandemic, following the FDA approval for COVID-19 vaccines and the USA Centres for Disease Control and Prevention (CDC) recommendations for vaccination, concerns about allergic reactions and vaccine safety have emerged among patients with pre-existing allergic conditions in the Middle East and worldwide, as well as concerns from other side effects (1). Allergic reactions to vaccines can be triggered by the vaccine, adjuvants, or excipients (2).

The incidence of severe allergic reactions secondary to the COVID-19 vaccines was low but somewhat higher than other vaccines. Reported reactions included anaphylaxis, acute urticaria, cutaneous, and delayed hypersensitivity reactions (3). The estimated incidence of anaphylaxis was approximately five cases per million doses of mRNA COVID-19 vaccines administered (2). Although reports were scarce in the Middle East, the incidence of allergic reactions secondary to the COVID-19 vaccine was low; symptoms ranged from skin rash to severe allergic reactions (4–7).

The exact mechanisms underlying these allergic responses and the role of allergy testing in managing such reactions are not yet fully understood. Excipients within the vaccine, such as polyethene glycol (PEG) and polysorbate 80, are suspected of playing a significant role in developing these reactions (8, 9).

PEG and polysorbate 80 are present in various medications and vaccines and have been associated with different types of allergic reactions, including anaphylaxis (10–12). In 2022, the ENDA/EAACI Position paper on COVID-19 vaccine allergy provided guidelines to help clinicians manage and offer safe recommendations to the public. The guideline recommended comprehensive clinical evaluation and allergy testing for patients with a history of immediate allergic reaction or anaphylaxis following COVID-19 vaccination, those with known allergies to vaccine components, and individuals with a history of recurrent drug-induced anaphylaxis. The recommended allergy testing includes skin prick tests (SPT), intradermal tests (ID), and patch tests for COVID-19 vaccine excipients, the vaccine itself, and potentially relevant allergens like latex and chlorhexidine (8). However, later, in 2023, an international consensus approach for evaluating and managing allergic reactions secondary to COVID-19 vaccines was published. The consensus recommended against allergy testing with the COVID-19 vaccine or its excipients to patients with or without a history of allergic reaction to the first dose of the vaccine (13). The USA CDC is still advising pre-vaccination screening to identify contraindications or a precaution to the immunisation and recommended consultation with an allergist when necessary (14).

There were several publications from different parts of the world about the allergic reactions to the COVID-19 vaccine, and researchers described their allergy evaluation approach (Supplementary Table E1), but, to our knowledge, no reports from the GULF region about this subject.

This report describes the frequency and type of allergic reactions following the mRNA COVID-19 vaccination, and it evaluates the use of allergy testing for PEG and polysorbate 80 excipients among patients who attended the adult allergy clinic at Hamad Medical Corporation (HMC), Doha, Qatar. Additionally, it offers valuable insights for healthcare providers to identify and manage potential allergic reactions, ultimately contributing to safer vaccination practices and reducing public concerns about vaccine safety.

## Materials and methods

This is a retrospective study that focused on patients who reported allergic reactions following COVID-19 vaccination at the adult Allergy and Immunology Division at HMC in Doha, Qatar, between April 2021 and August 2022 (population). These patients underwent supervised allergy skin testing for COVID-19 vaccine excipients (intervention). As this was a descriptive study, there was no comparison group. The outcome was the result of allergy testing and subsequent recommendations for future vaccinations.

Data collected included demographics, allergic comorbidities, allergic symptoms secondary to the mRNA COVID-19 vaccine, skin testing results, recommendations for further vaccinations, and the tolerability of subsequent COVID-19 vaccine doses in high-risk patients. The study received local ethical approval (MRC-04-24-589).

### Developing allergy skin testing during the pandemic

This test was developed during the pandemic to help local physicians and patients address fear and hesitancy around COVID-19 vaccination. The target population included adults with suspected allergic reactions to PEG and/or polysorbate 80 before their first mRNA vaccine, those who developed allergic reactions after the first or second dose of the mRNA vaccine, or those with personal concerns about receiving the vaccine because of a history of allergic conditions. For this report, we reviewed and analysed only those who developed allergic reactions after the mRNA COVID-19 vaccination.

The allergy skin testing targeted Polyethylene glycol (PEG3350) and polysorbate 80, utilising both SPT and ID testing with varying dilutions. Testing was conducted using drugs containing PEG3350 (Movicol, Methylprednisolone Acetate) and polysorbate 80 (Triamcinolone Acetonide, Prevnar 13). Negative skin tests were followed by intradermal testing with the same medications at different dilutions. We used Methylprednisolone sodium-succinate (solu-medrol) as negative control for SPT and ID and histamine and glycerinated saline as positive and negative controls for SPT (Table 1). The concentrations and dilutions used were based on previously published reports of non-irritant concentrations (8, 15).

**Table 1:** Component of the developed COVID-19 vaccine excipients test.

Excipient	Drug Name	Dilutions	Concentration
Skin Prick Test^a^			
PEG3350	Movicol (MiraLAX) (170 mg/ml)	1/100	1.7 mg/ml
		1/10	17 mg/ml
		1/1	170 mg/ml
	Methylprednisolone Acetate (Depo-Medrol)	1/1	40 mg/ml
Control	Methylprednisolone sodium-succinate (solumedrol)	1/1	40 mg/ml
Polysorbate 80	Triamcinolone Acetonide	1/1	40 mg/ml
	Prevnar 13	1/10	
Control	Histamine (positive control)		
	Glycerinated saline (negative control)		
Intradermal Tests^a^			
PEG3350	Methylprednisolone Acetate (Depo-Medrol)	1/100	0.4 mg/ml
		1/10	4 mg/ml
Control	Methylprednisolone sodium-succinate (solumedrol)	1/100	0.4 mg/ml
		1/10	4 mg/ml
Polysorbate 80	Triamcinolone Acetonide	1/100	0.4 mg/ml
		1/10	4 mg/ml
		1/1	40 mg/ml
	Prevnar 13	1/100	

^a^ During the testing period, all positive test results were compared against two healthy control subjects who were tested concurrently. Both control subjects consistently produced negative results, ensuring the accuracy of the positive findings.

SPT was conducted using a Single-use, single-site needle (Staller point). The test was considered positive if the patient developed a 3 mm or more wheal response after 15–20 min. The ID test was performed using a 27-gauge needle to inject 0.03 ml of the solution intradermally to form a bleb; an increase in the size of the initial bleb with erythema is considered a positive test (16).

All positive test results were compared against two healthy control subjects tested concurrently during the testing period. Both control subjects consistently produced negative results, ensuring the accuracy of the positive findings.

Before the procedure, a comprehensive medical history and physical examination were performed, and patients provided informed consent with venous access secured. The allergy skin testing was conducted in the allergy clinic under the supervision of an allergist, with resuscitation equipment readily available.

### Statistical analysis

Demographics and clinical characteristics of the patients were summarised using descriptive statistics. Categorical data were represented as frequency and percentage, while continuous data were expressed as mean ± standard deviation (SD) or median and interquartile range (IQR) as appropriate. The analysis was conducted using Microsoft Excel.

## Results

**Figure 1:**
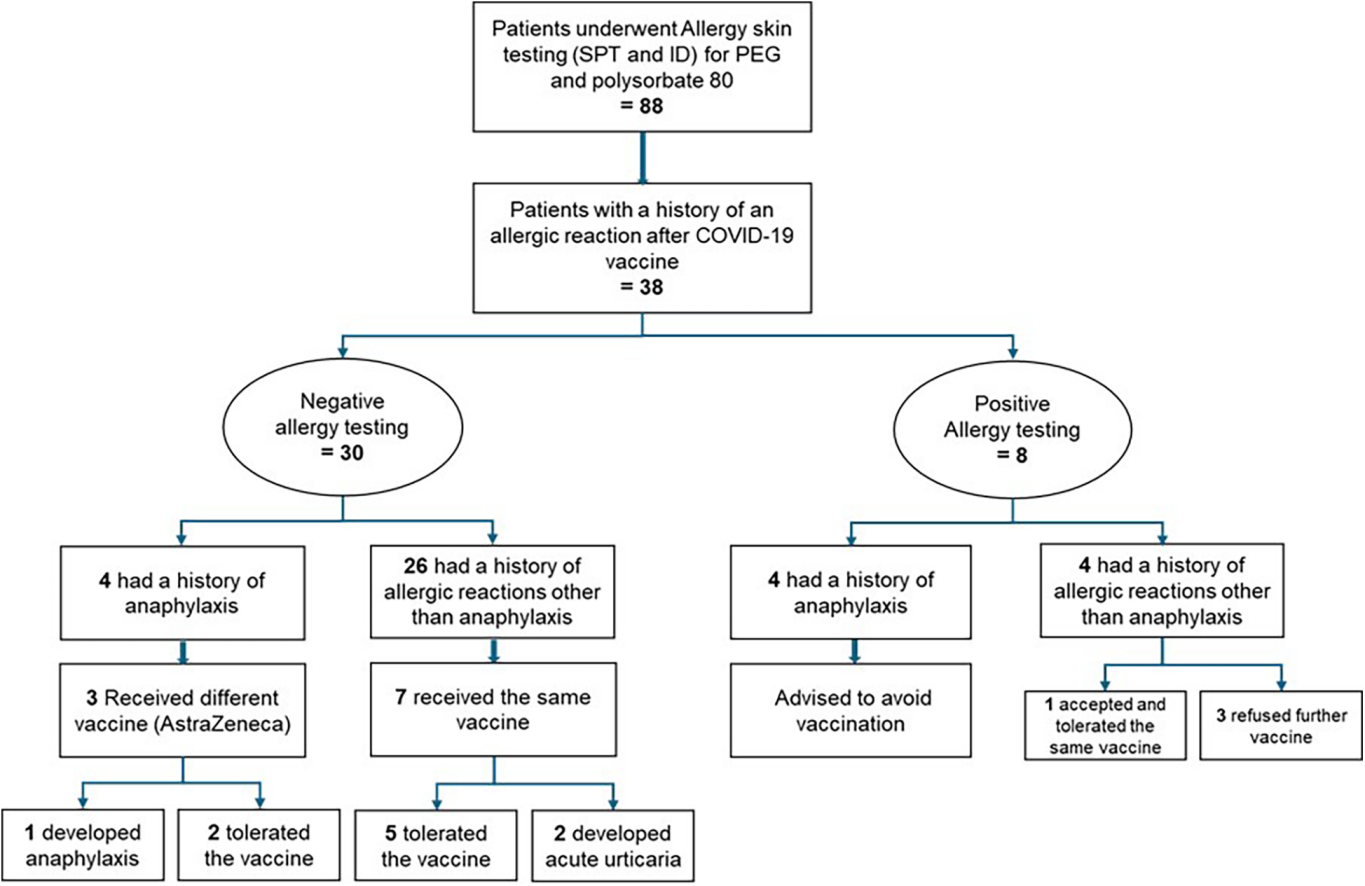
Flowchart of the study of COVID-19 excipients allergy skin testing and outcome.

A total of 88 patients underwent skin prick testing (SPT) and intradermal (ID) testing for COVID-19 vaccine excipients during the study period. Thirty-eight patients with a documented history of allergic reactions to mRNA COVID-19 vaccines met the inclusion criteria and were included in the final analysis. All patients completed both SPT and ID testing. The study flowchart is outlined in Figure 1.

### Demographic characteristics

The study population was predominantly female, representing 94.7% of the participants. The majority (*n* = 24, 63.2%) were of Arab ethnicity. The mean ± SD age of the patients was 41.26 ± 14.38 years. A large proportion (*n* = 27, 71.2%) had allergic comorbidities, with (*n* = 14, 36.8%) reporting drug allergies and (*n* = 7, 18.4%) reporting food allergies. A history of anaphylaxis related to insect, food or drugs was present in (*n* = 8, 21.1%) of the participants. Other common allergic comorbidities included asthma, allergic rhinitis, chronic urticaria, and atopic dermatitis (Table 2).

**Table 2:** Demographic characteristics and allergic comorbids of patients tested for COVID-19 excipients allergy.

Demographics	*N* = 38
Age (Year)	
Mean ± SD	41.26 ± 14.38
Median (IQR)	40 (49–28.25)
Range	19–75
Gender	
Female	36 (94.7%)
Ethnicity *n* (%)	
Arabs	24 (63.2%)
Caucasians	7 (18.4%)
Asians (non-Arabs)	7 (18.4%)
Allergic Comorbid^a^ *n* (%)	27 (71.2%)
Asthma	8 (21.1%)
Chronic spontaneous urticaria	7 (18.4%)
Allergic rhinitis	4 (10.5%)
Chronic rhinosinusitis	3 (7.9%)
Chronic Induced urticaria	1 (2.6%)
Atopic Dermatitis	1 (2.6%)
Drug allergy	14 (36.8%)
Penicillin	6 (15.8%)
NSAIDs	4 (10.5%)
Ciprofloxacin	1 (2.6%)
Lidocaine = 1	1 (2.6%)
Tamoxifen	1 (2.6%)
Can't remember	2 (5.3%)
Food Allergy	7 (18.4%)
Seafood	4 (10.5%)
Kiwi	1 (2.6%)
Mango	1 (2.6%)
Nuts	1 (2.6%)
Strawberry	1 (2.6%)
Insect allergy	2 (5.3%)
Previous Anaphylaxis	8(21.1%)

^a^ Some patients have more than one.

### The primary reaction to the mRNA COVID-19 vaccine

A history of anaphylaxis, as defined by the World Allergy Organization (WAO) and Brighton Collaboration Case Definition for Anaphylaxis (17), was documented in (*n* = 8, 21.1%) of patients following the first dose of mRNA COVID-19 vaccine (Table 3).

**Table 3:** Details of patients who developed anaphylaxis after the first dose of mRNA COVID-19 vaccine.

	Age (year)	Gender	Nationality	Allergic Comorbidity	Symptoms^a^	Brighton Collaboration criteria of anaphylaxis (17)	Allergy test result	Further vaccination
1	28	F	Indian	Asthma	Within 5 min: tongue swelling, shortness of breath and hypotension	Level 1	Negative	Received different vaccine and no reported reactions.
2	45	F	Irish	None	Within 20 min: itchy eyes, urticaria, shortness of breath, hypotension and dizziness	Level 1	Negative	No
3	51	M	Jordanian	Food allergy to Kiwi	Within 20 min: tongue and lips angioedema, urticaria, and loss of consciousness	Level 1	Negative	Received a different vaccine and developed urticaria, angioedema and shortness of breath
4	48	F	Qatari	None	Within 5 min: urticaria, itchy throat, vomiting and shortness of breath and wheezing	Level 1	Negative	Received different vaccine and no reported reactions.
5	28	F	Bahraini	CRS, Asthma, AD, CSU	Within 1 min: Urticaria, angioedema of the face and tongue, shortness of breath and dizziness.	Level 1	Positive ID test for polysorbate 80	No
6	29	F	Qatari	CRS, Asthma, insect allergy, Food Allergy (eggplant, kiwi, nuts and pineapple), Drug allergy (Lidocaine), CSU	within 4 h: Urticaria, facial angioedema, throat tightness and shortness of breath.	Level 1	Positive ID test for polysorbate 80	No
7	31	F	Qatari	Drug Allergy, Previous Anaphylaxis (after receiving Racecadotril and metronidazole)	Within 15 min: Urticaria, throat tightness and shortness of breath.	Level 2	Positive ID test for polysorbate 80	No
8	30	F	Saudi	Food allergy (seafood, strawberry, berries), Previous Anaphylaxis secondary to strawberry), Drug allergy (Augmentin)	Within 5 min: urticaria, angioedema of lips, shortness of breath and dizziness.	Level 1	Positive SPT for PEG and positive ID for Polysorbate 80	No

AD, atopic dermatitis; CRS, chronic rhinosinusitis; CSU, chronic sponatnoues urticaria.

^a^ Patient 1 received the Moderna vaccine. The remaining patients received the Pfizer vaccine.

The remaining 30 patients developed allergic reactions following the first dose (*n* = 26, 68.4%), the second dose (*n* = 3, 7.8%), and the third dose (*n* = 1, 2.6%) of the mRNA COVID-19 vaccine. The nature of the reactions was acute urticaria within 6 h in (*n* = 11, 28.9%) of cases, while a delayed urticarial rash appearing after 6 h was reported in (*n* = 6, 15.8%). Exacerbation of chronic urticaria was observed in (*n* = 3, 7.9%) patients. Other reactions included eczema in (*n* = 2, 5.3%), delayed exanthematous maculopapular rash, and erythematous macules in (*n* = 1, 2.6%) each. Additionally, (*n* = 6, 15.8%) of patients experienced nonspecific symptoms, such as pruritus without a visible rash, headache, and palpitation. The Pfizer-BioNTech BNT162b2 vaccine was administered to (*n* = 32, 84.2%) of the study population (Table 4).

**Table 4:** Allergic reactions related to mRNA COVID-19 vaccines and result of the COVID-19 vaccine excipient allergy skin testing.

COVID-19 vaccine Testing	Frequency (Percentage)
	*N* = 38
Allergic reaction from COVID-19 vaccine	
Acute Urticaria (within 6 h)	11 (28.9%)
Anaphylaxis	8 (21.1%)
Non-specific	6 (15.8%)
Urticaria acute (delayed after 6 h)	6 (15.8%)
Exacerbation of chronic urticrtia	3 (7.9%)
Eczema	2 (5.3%)
Delayed reaction exanthematous rash	1 (2.6%)
Acute non-itchy erythematous macules	1 (2.6%)
mRNA COVID-19 vaccine	
Pfizer-BioNTech BNT162b2	32 (84.2%)
Moderna mRNA-1273	6 (15.8%)
Positive Skin Prick test	2 (5.3%)
Polyethylene Glycol (PEG)	2 (5.3%)
Polysorbate 80	0 (0%)
Positive Intradermal test	7 (18.4%)
Polysorbate 80^a^	7 (18.4%)
PEG^a^	1 (2.6%)
Anaphylaxis developed during the test	1 (2.6%)
Second dose vaccine	11 (28.9%)
Received the same vaccine	8 (21.1%)
Received different vaccines (Pfizer, AstraZeneca)	3 (7.9%)
The outcome of those who received a second dose of the vaccine	11 (28.9%)
No reported reactions	8 (21.1%)
Developed similar reaction (anaphylaxis)	1 (2.6%)
Developed the same reaction (Acute urticaria)	2 (5.3%)

^a^ One patient has a positive Intradermal test for both PEG and polysorbate.

### Skin prick test (SPT) and intradermal (ID testing)

Eight patients (21.1%) were tested positive in our study.

One patient tested positive for medications containing PEG alone (Movicol) by SPT, while five patients tested positive for polysorbate 80-containing medications alone (Triamcinolone Acetonide), which was observed by ID testing. Another patient tested positive for both PEG (Movicol) by SPT and polysorbate 80 (Triamcinolone Acetonide) by ID testing. The last patient tested positive for both PEG (Methylprednisolone Acetate) and Polysorbate 80 (Prevnar 13) by ID testing.

Among the cohort of patients who reported anaphylaxis after receiving mRNA COVID-19 vaccine, four tested positive via ID.

Patient number 8 (Table 3) underwent allergy testing four months after the index reaction and had a positive SPT to PEG3350 1:1 of 170 mg/ml; we proceeded with the ID test for polysorbate 80 to find a safe alternative vaccine. She developed a positive ID test for triamcinolone acetonide at 1:1 of 40 mg/ml concentration. Ten minutes after the positive ID test and 40 min after the positive SPT, she developed generalized itching with urticarial rash, cough, shortness of breath, and a wheezy chest. She was treated with Epinephrine (1:1,000) 0.3 mg IM two injections 10 min apart, inhaled beta2-agonist, and hydrocortisone 200 mg IV. She was transferred to the ED for observation and was discharged after 10 h in stable condition. One week later, she was symptom-free during a clinic visit; spirometry showed an FEV1 of 67% predicted (2.20 L), with significant post-bronchodilator change, so the diagnosis of asthma was made at that point, and she started on regular inhaled corticosteroid. This patient was advised against COVID-19 vaccines.

### Impact of negative skin testing on subsequent COVID-19 vaccine doses

Of the 30 patients who tested negative for both SPT and ID testing, ten patients (33.3%) received an additional dose of the COVID-19 vaccine, while the remaining patients refrained from further doses. Seven patients received the same vaccine, two developed a similar reaction to the first dose, generalized urticaria, within a few minutes of receiving the vaccine, while the remaining five patients (71%) reported no further adverse reactions. The negative predictive value of skin testing was determined to be 71.4% (where true negatives were defined as patients with negative skin testing and who tolerated the vaccine).

Three patients (37.5%) of those who had previously experienced anaphylaxis after the first dose of the mRNA COVID-19 vaccine had negative allergy testing and received a different type of vaccine. Two (66%) tolerated the alternative vaccine without issues, while one (33%) experienced an anaphylactic reaction similar to the reaction triggered by the mRNA COVID-19 vaccine. This patient [patient number 3 (Table 3)] developed generalized urticaria, angioedema of the face and shortness of breath after receiving the AstraZeneca vaccine. He was treated in the emergency department with intramuscular epinephrine (Figure 1).

## Discussion

The reported allergic reactions, including severe cases, following the COVID-19 vaccination during the pandemic significantly contributed to vaccination hesitancy worldwide (17). The need for booster doses of the COVID-19 vaccine (14) prompted the development of a strategy to identify patients who can safely receive additional vaccinations and those who should avoid them despite the paucity of data on the outcome and validity of allergy testing. On the other hand, allergy consultation, with or without allergy testing, has helped to assure patients and reduce vaccination fear (18).

In addition, Banjri et al. proposed an algorithm to risk stratify patients based on their clinical history and allergist evaluation for the possibility of allergy testing (19). Therefore, utilizing available resources was crucial to address vaccine hesitancy during the pandemic.

Previous reports have shown that women are more likely than men to report adverse events following COVID-19 vaccination, with younger individuals experiencing a higher frequency of these reactions. At the same time, older adults are at greater risk for severe side effects (20). Our findings align with this, as most of our study population consisted of middle-aged females.

It has been proposed that individuals with food and drug allergies, along with a history of anaphylaxis, may have an elevated risk of developing cutaneous reactions to the COVID-19 vaccine; however, most of these reactions are generally mild (21). Our study observed a high prevalence of allergic comorbidities among our cohort, particularly food and drug allergies Furthermore, the majority of reported reactions in our cohort were mild.

Few studies have explored the procedure and outcome of allergy testing in individuals with a history of allergic reactions to COVID-19 vaccines or other allergic disorders from different populations (Supplementary Table E1). However, there were few reports from the Middle East and, to our knowledge, no reports from the Gulf region. Some researchers have also used COVID-19 vaccines for allergy testing. Most of the studies in Supplementary Table E1 concluded that the allergy skin testing for PEG and polysorbate 80 with or without the COVID-19 vaccine has limited predictive value in predicting tolerance to the second dose.

Few have concluded that the overall positivity rate of allergy skin testing has been low, but using the vaccine as part of the testing process may enhance sensitivity (22–24).

A 2023 systematic review and meta-analysis by Greenhawt, M. et al. analyzed 20 studies on skin testing (SPT) for BNT162b2, mRNA-1273, PEG, and Polysorbate 80. A total of 317 individuals underwent 578 skin tests. The overall sensitivity for predicting allergic reactions was 0.03 (95% CrI 0.01–0.08), with a specificity of 0.98 (95% CrI 0.95–1.00). Sensitivity for SPT with the mRNA vaccines was 0.2 (95% CrI 0.01–0.52) and specificity 0.97 (95% CrI 0.9–1). Sensitivity for PEG and Polysorbate 80 was lower, at 0.02 (95% CrI 0.00–0.07) and 0.03 (95% CrI 0.00–0.11), respectively, with specificity of 0.99 (95% CrI 0.96–1) for PEG and 0.97 (95% CrI 0.91–1) for Polysorbate 80 (25).

Due to limited resources and vaccine shortages during the early stages of the pandemic, our testing strategy focused on vaccine excipients rather than the vaccine itself. This approach was both resource-efficient and practical for addressing the challenges of that time. Also, to avoid false positive tests that were reported before with some medications due to local irritation effect (26), we performed the test on two healthy controls for each positive test. We calculated a negative predictive value of 71.4%, indicating that most patients with negative test results could safely receive the vaccine. However, this moderate predictive value highlights the need to remain cautious, as some risk of adverse reactions still exists.

Furthermore, the same meta-analysis reviewed three reports, revealing that most second-dose reactions occurred in non-sensitized individuals. Only a few patients had positive skin tests, with three reacting to the vaccine, one to PEG, and none to polysorbate 80. Among the six severe reactions following the second dose of an mRNA vaccine, four occurred in patients with no prior sensitisation to the vaccine or its excipients. The overall sensitivity of skin testing was notably low, identifying only around 3% of individuals who experienced immediate allergic reactions upon revaccination with the vaccine, PEG, or polysorbate 80 (25).

In our cohort study, 21.1% of the patients tested positive for vaccine excipients, and seven were advised to avoid further vaccination. However, one patient with mild initial symptoms could safely receive another dose of the same vaccine (Moderna) without complications despite having positive allergy ID testing for polysorbate 80 (Triamcinolone Acetonide). Of the 30 patients who tested negative, 10 (33.3%) patients only proceeded with further vaccinations. Seven (70%) received the same vaccine, while three patients opted for an alternative vaccine. Among these ten patients, three (30%) experienced a recurrence of the previous reaction (one developed anaphylaxis, and two had acute urticaria) and were advised against further doses, while the remaining patients reported no further reactions. No pre-medications or graded vaccine dosing were used for the subsequent vaccine doses. The remaining 20 patients who tested negative chose to abstain from receiving additional vaccine doses.

Interestingly, 50% of patients who reported anaphylaxis after receiving the first mRNA COVID-19 vaccine had positive test results, indicating that allergy testing might help to identify potential risks in patients with a history of immediate IgE-mediated reactions. However, the 50% rate also suggests that further studies are needed to refine these tests' predictive value and identify additional factors contributing to vaccine-related allergic reactions. Additionally, we could not revaccinate patients with positive test results due to safety and ethical considerations, limiting our ability to assess the test's positive predictive value.

Since August 2022, we have stopped performing allergy skin testing for the COVID-19 vaccine excipients. After allergy consultation, anxious patients or patients suspected to have an allergy to PEG or polysorbate 80 were referred to administer the vaccine under supervision at the HMC Center of Communicable Disease (i.e., in a hospital setting) or advised to receive a vaccine not containing PEG or polysorbate 80 based on availability and clinical suspicion.

To our knowledge, this is the first study from the Gulf region to report the frequency and type of allergic reactions to the COVID-19 vaccine and the use of COVID-19 vaccine excipients allergy testing. A limitation of this study was its retrospective nature, and the testing did not include the COVID-19 vaccine itself due to limited accessibility and resources of the COVID-19 vaccine during the pandemic. Another limitation was a possible underestimation of patients tolerating further vaccinations because most patients with negative allergy testing refused to receive further vaccine doses.

## Conclusion

Most allergic reactions secondary to the mRNA COVID-19 vaccine reported in Qatar were mild. During the pandemic, a specific management protocol for patients with a history of allergic reactions to the COVID-19 vaccine was required to assure the public and reduce vaccine hesitancy. Our test showed a low positivity rate. Based on current evidence, we acknowledge that routine skin testing with PEG or polysorbate 80 may not help predict or risk-stratify patients for COVID-19 vaccination decisions. Further research is needed to explore and understand the exact mechanism of COVID-19 vaccine allergic reactions, hence developing an accurate allergy testing protocol.

## Data Availability

The original contributions presented in the study are included in the article/Supplementary Material, further inquiries can be directed to the corresponding author.

## References

[1] Dadras O SeyedAlinaghi S Karimi A Shamsabadi A Mahdiabadi S Mohammadi P Public acceptability of COVID-19 vaccines and its predictors in Middle Eastern/North African (MENA) countries: a systematic review. Hum Vaccines Immunother. (2022) 18(5):2043719. 10.1080/21645515.2022.2043719 35318872 PMC9196809

[2] Klein NP Lewis N Goddard K Fireman B Zerbo O Hanson KE Surveillance for adverse events after COVID-19 mRNA vaccination. JAMA. (2021) 326(14):1390. 10.1001/jama.2021.15072 34477808 PMC8511971

[3] Paul P Janjua E AlSubaie M Ramadorai V Mushannen B Vattoth AL Breathless encounters: analyzing anaphylaxis at the crossroads of COVID-19 vaccination. Qatar Med J. (2024) 2024(2):9. 10.5339/qmj.2024.qitc.9 38680410 PMC11046092

[4] Almohaya AM Alsubie H Alqarni B Alzayad B Alghar A Alshahrani K Acute unsolicited adverse events following BNT162b2 vaccine in Saudi Arabia, a real-world data. Vaccine. (2022) 40(3):477–82. 10.1016/j.vaccine.2021.12.001 34916104 PMC8668155

[5] Hatmal MM Al-Hatamleh MAI Olaimat AN Hatmal M Alhaj-Qasem DM Olaimat TM Side effects and perceptions following COVID-19 vaccination in Jordan: a randomized, cross-sectional study implementing machine learning for predicting severity of side effects. Vaccines. (2021) 9(6):556. 10.3390/vaccines9060556 34073382 PMC8229440

[6] Murished GM Dandachi I Aljabr W. Side effects of COVID-19 vaccines in the middle eastern population. Front Immunol. (2023) 14:1270187. 10.3389/fimmu.2023.1270187 38022593 PMC10654979

[7] Alhossan A Alsaran AK Almahmudi AH Aljohani ZS Albishi MR Almutairi AK. Adverse events of COVID-19 vaccination among the Saudi population: a systematic review and meta-analysis. Vaccines. (2022) 10(12):2089. 10.3390/vaccines10122089 36560499 PMC9783010

[8] Barbaud A Garvey LH Arcolaci A Brockow K Mori F Mayorga C Allergies and COVID-19 vaccines: an ENDA/EAACI position paper. Allergy. (2022) 77(8):2292–312. 10.1111/all.15241 35112371

[9] Viggiani F Calogiuri G Paolino D Griscti Soler D Pugliese F Zaza I Predictive value of skin testing with excipients for COVID-19 vaccines. Explor Asthma Allergy. (2024) 2:49–64. 10.37349/eaa.2024.00028

[10] Wenande E Garvey LH. Immediate-type hypersensitivity to polyethylene glycols: a review. Clin Experimental Allergy. (2016) 46(7):907–22. 10.1111/cea.12760 27196817

[11] Co-Minh HB Demoly P Guillot B Raison-Peyron N. Anaphylactic shock after oral intake and contact urticaria due to polyethylene glycols. Allergy. (2007) 62(1):92–3. 10.1111/j.1398-9995.2006.01265.x 17156356

[12] Palacios Castaño M Venturini Díaz M Lobera Labairu T González Mahave I Del Pozo Gil M Blasco Sarramián A. Anaphylaxis due to the excipient polysorbate 80. J Investig Allergol Clin Immunol. (2016) 26(6):394–6. 10.18176/jiaci.0109 27996954

[13] Greenhawt M Dribin TE Abrams EM Shaker M Chu DK Golden DBK Updated guidance regarding the risk of allergic reactions to COVID-19 vaccines and recommended evaluation and management: a GRADE assessment and international consensus approach. J Allergy Clin Immunol. (2023) 152(2):309–25. 10.1016/j.jaci.2023.05.019 37295474 PMC10247143

[14] Centers for Disease Control and Prevention. “Interim Clinical Considerations for Use of COVID-19 Vaccines Currently Approved or Authorized in the United States.” Available online at: https://www.cdc.gov/vaccines/covid-19/clinical-considerations/interim-considerations-us.html#table-03 (accessed August 2024).

[15] Banerji A Wickner PG Saff R Stone CA Robinson LB Long AA mRNA vaccines to prevent COVID-19 disease and reported allergic reactions: current evidence and suggested approach. J Allergy Clin Immunol. (2021) 9(4):1423–37. 10.1016/j.jaip.2020.12.047 PMC7948517 33388478

[16] Ansotegui IJ Melioli G Canonica GW Caraballo L Villa E Ebisawa M Ige allergy diagnostics and other relevant tests in allergy, a World Allergy Organization position paper. World Allergy Organ J. (2020) 13(2):100080. 10.1016/j.waojou.2019.100080 32128023 PMC7044795

[17] Cardona V Ansotegui IJ Ebisawa M El-Gamal Y Fernandez Rivas M Fineman S World allergy organization anaphylaxis guidance 2020. World Allergy Organ J. (2020) 13(10):100472. 10.1016/j.waojou.2020.100472 33204386 PMC7607509

[18] Pindel T Brandstetter S Sieber W Kabesch M. Allergy skin prick tests with COVID-19 vaccines and their contribution to improve vaccination readiness and reduce anxiety. Allergo J Int. (2024) 33(5):153–8. 10.1007/s40629-024-00296-7

[19] Banerji A Wolfson AR Wickner PG Cogan AS McMahon AE Saff R COVID-19 vaccination in patients with reported allergic reactions: updated evidence and suggested approach. J Allergy Clin Immunol. (2021) 9(6):2135–8. 10.1016/j.jaip.2021.03.053 PMC8049186 33866033

[20] Yin A Wang N Shea PJ Rosser EN Kuo H Shapiro JR Sex and gender differences in adverse events following influenza and COVID-19 vaccination. Biol Sex Differ. (2024) 15(1):50. 10.1186/s13293-024-00625-z 38890702 PMC11184791

[21] Teh HL Keowmani T Tang MM. Risk factors associated with cutaneous reactions following COVID-19 vaccine immunisation: a registry-based case-control study. Malays J Med Sci. (2024) 31(3):133–48. 10.21315/mjms2024.31.3.10 38984235 PMC11229573

[22] Shavit R Maoz-Segal R Offengenden I Yahia SH Maayan DM Lifshitz Y Assessment of immediate allergic reactions after immunization with the Pfizer BNT162b2 vaccine using intradermal skin testing with the COVID-19 vaccines. J Allergy Clin Immunol. (2022) 10(10):2677–84. 10.1016/j.jaip.2022.08.010 PMC9375246 35973526

[23] Svarca L Bojadzieva S Rashiti P Rashiti Bytyci A. Allergic reactions with skin prick test and intradermal test from the anti COVID 19 vaccines at patients with high risk for hypersensitivity our experience. J Morphol Sci. (2024) 7(1):67–76. 10.55302/JMS2471067s

[24] Pitlick MM Sitek AN D’Netto ME Dages KN Chiarella SE Gonzalez-Estrada A Utility and futility of skin testing to address concerns surrounding messenger RNA coronavirus disease 2019 vaccine reactions. Ann Allergy Asthma Immunol. (2022) 128(2):153–60. 10.1016/j.anai.2021.11.006 34798275 PMC8594060

[25] Greenhawt M Shaker M Golden DBK Abrams EM Blumenthal KG Wolfson AR Diagnostic accuracy of vaccine and vaccine excipient testing in the setting of allergic reactions to COVID -19 vaccines: a systematic review and meta-analysis. Allergy. (2023) 78(1):71–83. 10.1111/all.15571 36321821 PMC9878056

[26] Wolfson AR Robinson LB Li L McMahon AE Cogan AS Fu X First-Dose mRNA COVID-19 vaccine allergic reactions: limited role for excipient skin testing. J Allergy Clin Immunol. (2021) 9(9):3308–3320.e3. 10.1016/j.jaip.2021.06.010 PMC8217699 34166844

