# Supplementary material for: Challenges in diagnosing polyethylene glycol and polysorbate 80 allergies: implications for allergic reactions in COVID-19 mRNA vaccination program: experience from Qatar
Source: Front Allergy. 2025 Jan 7;5:1502285. doi: 10.3389/falgy.2024.1502285 (PMC11753240; doi:10.3389/falgy.2024.1502285)
Supplement: Supplementary file 1 [file Table1.docx]

Table E1: Studies reported Allergy skin testing for COVID-19 vaccine and its excipients:

| Study, year of publication, country | Study methodology and time (month/year) | Female % (NO/Total No of patients) | Patients reported reaction to COVID-19 vaccine (NO/%) | Medications used for polysorbate 80 testing | Medications used for PEG testing | COVID-19 Vaccine used in testing | Patients with positive allergy testing (NO/%) | Patients with positive allergy testing reacted second dose of the same or different vaccine | Patients with positive allergy testing tolerate second dose of the same or different vaccine | Conclusion |
| --- | --- | --- | --- | --- | --- | --- | --- | --- | --- | --- |
| Wolfson et al. 2021, USA  (1) | Prospective, January to March 2021 | 89% (71/80) | 80 (100%) | Triamcinolone acetonide, Refresh Tears | Miralax, Methylprednisolone acetate | No | 14 (18%) | 30% (3/10) | 70% (7/10) | Skin testing has little utility in assessing non-anaphylaxis allergic reactions secondary to COVID-19 vaccine |
| ALMuhizi et al. 2022, Canada  (2) | January 1, to October 31, 2021 | 88.7% (39/44) | 40 (91%) + 4 with previous PEG-related allergies | Polysorbate 80 (Tween80) | PEG 300, PEG 3350, PEG 3000, PEG 20,000 | No | -7.5%  (3/40)  - 50%  (2/4) | 33% (1/3) non-severe reaction | -66%  (2/3)  -100%  (2/2) tolerated different vaccine | PEG testing is often unnecessary; most patients tolerated vaccination |
| Otani et al. 2022, USA  (3) | December 1, 2020, to August 31, 2021 | 89%  39/44 | 44 (100%) | Triamcinolone acetate, Prevnar-13 | Miralax, Methylprednisolone acetate | N0 | (3/14) 21%  questionable positives | 0/3 | 3/3 | Skin testing had limited predictive value for second-dose tolerance. |
| Svarca et al. 2024, Kosovo  (4) | December 2020 to February 2023 | 69.6% (163/234) | 234 (high-risk/allergy history | Not specified | Not specified | 66% (155/234)  (BNT162b2, Pfizer), 34%  (79/234) AZD1222, AstraZeneca | (4/155) 2.58% for Pfizer, (3/79) 3.8% for AZ | - | - | Most reactions to COVID  19 vaccinations are mild, self-limiting, and did not discourage vaccination |
| Vidal Oribe et al. 2022, Spain  (5) | 2022, Case report | Not specified | 5 | polysorbate 80 | PEG 4000 (Casenlax), PEG 3350 (Movicol)  PEG 2000, PEG 1500 (Roxall) | Pfizer, Moderna, AstraZeneca | 1 positive IDT with PS80 and with all the vaccines | 0/1 | 0/1 | Patients allergic to polysorbate 80 may tolerate PEG-containing vaccines. |
| Pitlick et al. 2022, USA  (6) | Retrospective.  January to July 2021 | Cohort 1:(44/55) 80%  Cohort 2: 65/74 (87.8%) | Cohort 1: 55 | Triamcinolone acetonide, Prevnar-13 | Miralax, fresh polysorbate compounds | Pfizer-BioNTech, Janssen in 11 patients | Cohort 1  (4/55) 7.3%  Cohort 2: (8/74) 10.8% | Cohort 1: 25% (1/4)  Cohort 2: 0/8 | Cohort 1: 50% (2/4)  Cohort 2:  37.5% (3/8) | Skin testing showed limited utility; graded dosing often effective. |
| Shavit et al. 2022, Israel  (7) | March to December 2021 | 92.1% (47/51) | 51 | - | PEG-3350, Normalax, methylprednisolone acetate | Pfizer (BNT162b2), AstraZeneca (AZD1222) | (6/51) 11.7% | 16.6% (1/6) reacted to different vaccine | 83%  (5/6) | Vaccine IDT with either BNT162b2 or AZD1222 vaccine may identify sensitized patients. |

References:

1. Wolfson AR, Robinson LB, Li L, McMahon AE, Cogan AS, Fu X, et al. First-Dose mRNA COVID-19 Vaccine Allergic Reactions: Limited Role for Excipient Skin Testing. The Journal of Allergy and Clinical Immunology: In Practice. 2021 Sep;9(9):3308-3320.e3.

2. ALMuhizi F, Fein M, Gabrielli S, Gilbert L, Tsoukas C, Ben-Shoshan M, et al. Allergic reactions to the coronavirus disease 2019 vaccine (ARCOV) study. Annals of Allergy, Asthma & Immunology. 2022 Aug;129(2):182-188.e1.

3. Otani IM, Tsao LR, Tang M. Coronavirus disease 2019 vaccine administration in patients with reported reactions to polyethylene glycol- and polysorbate-containing therapeutics. Annals of Allergy, Asthma & Immunology. 2022 Jul;129(1):88-94.e1.

4. Svarca L, Bojadzieva S, Rashiti P, Rashiti Bytyci A. ALLERGIC REACTIONS WITH SKIN PRICK TEST AND INTRADERMAL TEST FROM THE ANTI COVID 19 VACCINES AT PATIENTS WITH HIGH RISK FOR HYPERSENSITIVITY OUR EXPERIENCE. JMS. 2024;7(1):67–76.

5. Vidal Oribe I, Venturini Díaz M, Hernández Alfonso P, Del Pozo Gil M, González Mahave I, Lobera Labairu T. Tolerance to SARS CoV-2 Vaccines Containing Polyethylene Glycol in Patients Allergic to Polysorbate 80. J Investig Allergol Clin Immunol. 2022 Oct 10;32(5):403–5.

6. Pitlick MM, Sitek AN, D’Netto ME, Dages KN, Chiarella SE, Gonzalez-Estrada A, et al. Utility and futility of skin testing to address concerns surrounding messenger RNA coronavirus disease 2019 vaccine reactions. Annals of Allergy, Asthma & Immunology. 2022 Feb;128(2):153–60.

7. Shavit R, Maoz-Segal R, Offengenden I, Yahia SH, Maayan DM, Lifshitz Y, et al. Assessment of Immediate Allergic Reactions After Immunization With the Pfizer BNT162b2 Vaccine Using Intradermal Skin Testing With the COVID-19 Vaccines. The Journal of Allergy and Clinical Immunology: In Practice. 2022 Oct;10(10):2677–84.
